# Pharmacological Evaluation of Cannabinoid Receptor Modulators Using GRAB_eCB2.0_ Sensor

**DOI:** 10.3390/ijms25095012

**Published:** 2024-05-03

**Authors:** Samay Shivshankar, Josephine Nimely, Henry Puhl, Malliga R. Iyer

**Affiliations:** 1 Section on Medicinal Chemistry, National Institute on Alcohol Abuse and Alcoholism, National Institutes of Health, 5625 Fishers Lane, Rockville, MD 20852, USA; 2 Laboratory of Biophotonics and Quantum Biology, National Institute on Alcohol Abuse and Alcoholism, National Institutes of Health, 5625 Fishers Lane, Rockville, MD 20852, USA; puhlh@mail.nih.gov

**Keywords:** GRABeCB2.0, 2-AG, CB1R, CB2R, AEA, allosteric, orthosteric

## Abstract

Cannabinoid receptors CB_1_R and CB_2_R are G-protein coupled receptors acted upon by endocannabinoids (eCBs), namely 2-arachidonoylglycerol (2-AG) and *N*-arachidonoyl ethanolamine (AEA), with unique pharmacology and modulate disparate physiological processes. A genetically encoded GPCR activation-based sensor that was developed recently—GRAB_eCB2.0_—has been shown to be capable of monitoring real-time changes in eCB levels in cultured cells and preclinical models. However, its responsiveness to exogenous synthetic cannabinoid agents, particularly antagonists and allosteric modulators, has not been extensively characterized. This current study expands upon the pharmacological characteristics of GRAB_eCB2.0_ to enhance the understanding of fluorescent signal alterations in response to various functionally indiscriminate cannabinoid ligands. The results from this study could enhance the utility of the GRAB_eCB2.0_ sensor for in vitro as well as in vivo studies of cannabinoid action and may aid in the development of novel ligands.

## 1. Introduction

The endocannabinoid system (ECS) is a sophisticated mechanism comprising signaling pathways and endogenous modulators that intricately regulate various processes in the human body [1]. Specifically, endocannabinoids (eCBs) such as 2-arachidonoylglycerol (2-AG) and *N*-arachidonoyl ethanolamine (AEA), interact with the G-protein coupled cannabinoid receptors CB_1_ and CB_2_, activating them in distinct ways and influencing various signaling processes uniquely [2,3]. Notably, these same receptors recognize the active component of marijuana, Δ^9^-tetrahydrocannabinol (THC). Both CB_1_ and CB_2_ receptors play a crucial role in various physiological and pathophysiological processes in the central nervous system and peripheral tissues [4]. CB_1_ receptors are prominently expressed in the brain, where they are one of the most highly expressed GPCRs, and at lesser yet significantly operative levels in peripheral tissues like the lung, liver, and kidney [5]. CB_2_ receptors, on the other hand, are mainly found in immune cells as well as hematopoietic systems, although brain CB_2_ receptors have also been extensively documented [5,6,7].

The primary intracellular regulators activated by CB_1_R are heterotrimeric G proteins from the G_(i/o)_ family. The G-protein subunits inhibit adenylyl cyclase while also modulating ion channels, including those for calcium and potassium ions [8]. Activation of CB_1_R additionally results in the phosphorylation and activation of mitogen-activated protein kinases (MAPK), such as p42/p44 MAPK, p38 MAPK, and c-Jun N-terminal kinase, which have the capacity to regulate nuclear transcription factors [8]. Furthermore, activated and phosphorylated CB_1_R form associations with β-arrestin, leading to complexes which can contribute to receptor desensitization and internalization [9]. The expansive reach of CB_1_R across the human body and the multitude of physiological pathways in which it participates make it a strong candidate for pharmaceutical targeting [10].

The endocannabinoid/CB_1_R system (ECS) is a crucial regulator of lipid and carbohydrate metabolism [11,12]. CB_1_R activation promotes energy conservation and inhibits energy expenditure, with an overactive ECS contributing to the development of visceral obesity and the associated metabolic syndrome [13,14,15]. CB_1_R antagonists show promising anti-obesity effects and cardiometabolic improvements in obese subjects, yet the clinical utility of the receptor has not been harnessed beyond the now-withdrawn agent Acomplia (rimonabant) due to unacceptable neuropsychiatric side effects [16,17]. Hence, tools and probes advancing the understanding of the CB_1_R-based pathway hold great promise to develop safer therapeutics based on this mechanism of action (MoA) [15,18,19].

To monitor real-time fluctuations in eCB lipid mediator levels in cultured cells and rodent models acting through CB_1_R, an elegant tool in the form of a genetically encoded fluorescent sensor named GRAB_eCB2.0_ has been developed [20]. Its activation by eCB analogs produced by cells and limited phytocannabinoids has also now been characterized [21]. This characterization is crucial for enhancing the tool’s effectiveness in studying the pharmacological actions of cannabinoids in vivo. However, many CB_1_R ligands and would-be therapeutics are exogeneous, lipophilic, synthetic ligands belonging to numerous different chemotypes whose ability to interact with GRAB_eCB2.0_ is still unclear. Understanding the interaction and binding dynamics of these probes could offer additional utility for GRAB_eCB2.0_ and opportunities to improve the sensor technology broadly for in vitro and in vivo pharmacological application [22,23,24]. In the current study we sought to analyze the actions of various classes of synthetic cannabinoid ligands on the GRAB_eCB2.0_ sensor. The results obtained here present a window into utilizing the CB_1_R-based GRAB_eCB2.0_ for the rapid assessment of apparent binding affinities and preliminary functional characterization of tested ligands. While the sensor is predominantly activated by compounds that act on the receptor as agonists, we have now developed a robust method for measuring apparent binding affinities of compounds that do not inherently activate the sensor but behave as CB_1_ functional antagonists/inverse agonists.

As a standard GPCR, the effects of eCBs and pharmaceutical ligands for drug discovery are measured using many standardized assays on CB_1_Rs. These assays include radioligand binding, GTPγS, cAMP, β-arrestin, BRET-based assays, and many more [25]. These assays work to discover binding affinities and the activity of the receptor in the presence of different modulators. These assays can, at times, pose challenges when dealing with radioisotopes, sophisticated instruments, and convoluted protocols, along with prohibitively expensive assay reagents. Li and co-workers have developed the novel GRAB_eCB2.0_ sensor, engineered from CB_1_R, by incorporating a circularly permutated-green fluorescent protein (cpGFP) in intracellular loop 3 (ICL3), where the native receptor interacts with heterotrimeric G-proteins [20]. Through iterative approaches, the sensor was shown to be a fast, reliable, cell-based tool that can qualitatively divulge activity and apparent binding of eCBs and modulators of the CB_1_R. This sensor works via the conformational change caused in the transmembrane domain when a modulator binds and causes the fluorescence of GFP to change. This sensor lacks the functional signaling domains of the receptor and allows for measurement through the visualization of the increased fluorescence upon binding to the sensor by way of activation. As developed, the sensor has been shown to exhibit robust fluorescence increases when exposed to the eCBs AEA and 2-AG in cultured neural cells, acute brain tissue slices, and targeted brain tissues in vivo, such as the amygdala and hippocampus. In cultured HEK293 cells, live cell confocal microscopy and fluorescent signal measurements were used to test many eCBs, phytocannabinoids, and prototypical CB_1_R antagonist SR141617A (rimonabant) by Stella and coworkers [21].

The GRAB_eCB2.0_ sensor has been proven to help detect eCBs and exogenous ligands. As an extension, in the current study, we validate the sensor’s performance to detect synthetic agonists and now show that the sensor-based measurements allow for an expedient assay that can uncover antagonist potencies of CB_1_R modulators based on its ability to displace agonists. We also tested known and reported allosteric modulators, both positive and negative, for effects on the sensor. Branching this method towards novel compounds will allow one to conduct a fast, reliable assay that can ascertain qualitatively whether a modulator behaves as an agonist or antagonist.

## 2. Results and Discussion

### 2.1. GRAB_eCB2.0_ Assays for CB1R Agonists

**Figure 1:**
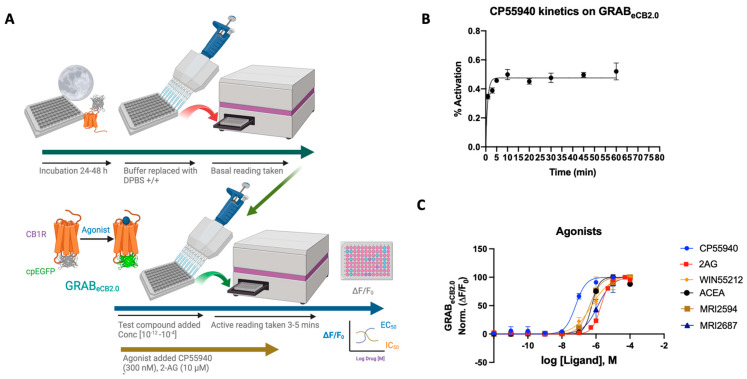
(**A**) Schematic depiction of the assay principle of the reported GRAB_eCB2.0_ sensor optimized in HEKtsA201 cells. Agonist binding activates the sensor, inducing a change in fluorescence. (**B**) Kinetics graph of 300 nM CP55940 run at varying incubation time periods of 1, 3, 5, 10, 20, 30, 45, and 60 min. Plateau occurs after 5 min with limited deviation from the normal. Optimum time points for reading between 4 and 7 min. (**C**) Concentration-dependent responses and EC_50_s of 2-AG, ACEA, CP55940, WIN55212-2, MRI-2594, and MRI-2687 at inducing GRAB_eCB2.0_ fluorescent signal, as determined by averaging ΔF/F_0_ normalized between 4 and 5 min (*vide infra*). EC_50_ values were calculated by GraphPad Prism 9. Data represent mean ± SEM from minimum three independent experiments.

Agonists of the CB_1_ receptor have been shown to have therapeutic effects in various animal models and clinical trials [18]. CB1 receptor agonists work as an antinociceptive to combat acute and chronic pain, reduce symptoms of stress and anxiety, and elevate symptoms of withdrawal in chronic drug-dependent patients for long-term alcohol, cannabis, and opiate use [26]. Yet, psychotropic properties limit the clinical use of many cannabinoid agonists [27]. The GRAB_eCB2.0_ sensor was initially designed to look at ligand dynamics of endocannabinoids that are part of the ECS and thus, upon ligand binding, show concentration-dependent increase in fluorescence. Validating previous work, we show that synthetic, exogenous agonists as well as components of the ECS like 2-AG and AEA respond to the sensor as reported [20,21] (see Figure 1A,B, Section 3).

2-AG is a naturally occurring endocannabinoid with a relatively high concentration in the central nervous system [28]. Using the GRAB_eCB2.0_ sensor in HEK293 cells, an EC_50_ of ~2 μM was obtained for 2-AG, showing an increase in fluorescence upon ligand binding. This is in line with reported data using the GRAB_eCB2.0_ sensor, delivering an EC_50_ for 2-AG at a value of 3.1 μM [20]. The reported EC_50_ of 2-AG by Stella’s group was 85 nM [21] (Figure 1C).

CP-55,940 is a strong CB_1_ receptor full agonist with a reported binding affinity 45 times that of Δ^9^-tetrahydrocannabinol (THC), the active compound found in marijuana [29,30]. CP-55,940 has also been published to have therapeutic effects as both an antinociceptive and an antiemetic [31]. However, reported adverse effects of CP-55,940 preclude its use as a therapeutic [32]. As a potent CB_1_ receptor agonist, the reported EC_50_ of CP-55,940 ranges from the sub-nanomolar value of 0.04 nM to 31 nM. The GRAB_eCB2.0_ sensor under our reported conditions delivered an apparent EC_50_ of 64 ± 8 nM.

WIN55212-2 is a potent full agonist of the CB_1_ receptor and has been used in experimental as well as clinical studies [33,34,35,36]. Mice treated with WIN55212-2 have been shown to have a significant reduction in pain, making it a plausible analgesic [37]. Clinical studies using WIN55212-2 have shown that the modulator can reduce interocular pressure in patients with glaucoma resistant to other therapies [34]. Reportedly, WIN55212-2 has an EC_50_ ranging from 5.5 nM to 3000 nM [37]. Using the sensor in HEK cells, an EC_50_ of 564 ± 34 nM was achieved in the range of reported values. As tested previously, the plot displays a strong agonist curve, giving both qualitative and quantitative validity and reported for EC_50_ [20].

ACEA is a CB_1_ selective agonist that shares much of its chemical structure with AEA, an endocannabinoid produced in the central nervous system. ACEA has been shown to bind (Ki = 1.5–5 nM) with a reported EC_50_ of 51 nM for the CB_1_ receptor [38]. It has also been used for its therapeutic effects on the ECS as an antidepressant and nociceptive, and it works as an anticonvulsant in patients with epilepsy [38]. ACEA on the GRAB_eCB2.0_ sensor functions as an agonist, but with a diminished EC_50_ of 572 ± 4 nM. We also assayed CB_2_R agonist/antagonist pair MRI-2594 and MRI-2687 to test their CB_1_ binding activity via their ability to modulate the sensor fluorescence [39]. As reported, the compounds indeed show weak CB_1_ agonism upon sensor binding, where EC_50_ = 527 ± 27 nM and 1.3 μM, respectively.

In all cases tested, the agonists activated the sensor, increased the fluorescence and valid EC_50_s could be obtained. It should be noted that the agonists for GRAB_eCB2.0_ expressed in HEK293 cells activated the sensor differentially, possibly indicating differing ligand dynamics. Altogether the sensor offers a viable tool that can be activated concentration-dependently and used to determine the EC_50_ of the ligand upon binding to the sensor, the EC_50_ values obtained in general varied to a significant extent (at least 10×) in comparison to the literature-reported values obtained using wild-type CB_1_R or membrane preparation.

### 2.2. GRAB_eCB2.0_ Assays for Antagonist/Inverse Agonists

Antagonists of the CB_1_R have been sought for their potential beneficial effects in obesity, metabolic disorders, fibrosis, and drug dependence [15,40]. Previous work with the sensor has shown both rimonabant and AM251 decrease sensor fluorescence [20]. Stella and coworkers reported that rimonabant, decreased GRAB_eCB2.0_ signal responses with a reported IC_50_ value of 3.3 nM, mirroring its reported potency for CB_1_R [21]. We sought to investigate the responses of various CB_1_ antagonists belonging to differing chemotypes on the GRAB_eCB2.0_ sensor. Prototypical CB_1_ antagonists are classified as 3-arm bearing compounds. CB_1_ antagonists like rimonabant, taranabant, and otenabant belong to the 3-arm class based on their orientation in the inactive binding of CB_1_ in the crystal structure [41].

### 2.3. 3-Arm Modulators

**Figure 2:**
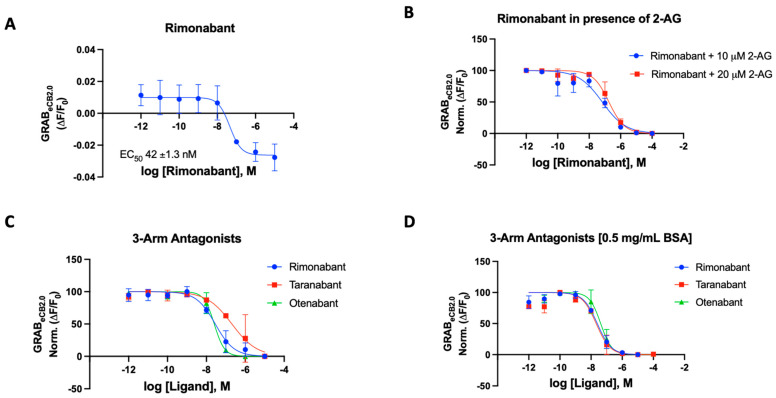
(**A**) Fluorescent signal of rimonabant at inhibiting GRAB_eCB2.0_ basal fluorescence as determined by averaging ΔF/F_0_ between 4 and 5 min. (**B**) Antagonist potency of rimonabant at inhibiting GRAB_eCB2.0_ fluorescence in presence of 10 mM and 20 mM of 2-AG, as determined by averaging ΔF/F_0_ normalized between 4 and 5 min. (**C**) Antagonist potency of 3-arm ligands at inhibiting GRAB_eCB2.0_ fluorescence in presence of 300 nM of CP55940 and in absence of BSA, as determined by averaging ΔF/F_0_ normalized between 4 and 5 min. (**D**) Antagonist potency of 3-arm ligands at inhibiting GRAB_eCB2.0_ fluorescence in presence of 300 nM of CP55940 with 0.5 mg/mL BSA, as determined by averaging ΔF/F_0_ normalized between 4 and 5 min. IC_50_ values were calculated by GraphPad Prism 9. Data represent mean ± SEM from minimum three independent experiments (Table 1).

With a standardized procedure in hand, we validated rimonabant’s behavior on the GRAB sensor. The literature-reported potencies for rimonabant range from an IC_50_ of 5.6 nM to 48 nM [42]. The treatment of rimonabant on the GRAB_eCB2.0_ sensor stable HEK293 cells showed an inhibition of fluorescence and below baseline level indicative of inverse agonism (measured EC_50_ = 42 ± 3 nM, Figure 2A). This is in line with reported data for rimonabant by Stella and co-workers. However, in mimicking the antagonist functional assay, the sensor could be activated by agonists like CP559450 or 2-AG. The CP559450 quench by an antagonist could be concentration-dependently observed by decreased fluorescence showing antagonism and producing a potent CB_1_ antagonist curve for rimonabant in the same experimental system. An area of concern was the lipophilicity of the ligands tested in the assay. The HEK293 cells are kept viable in the 96-well plate with DPBS buffer. The DPBS buffer poses challenges in dissolving highly lipophilic cannabinoid agents. To combat this problem, optimum inclusion of bovine serum albumin (BSA) was tested and 0.5 mg/mL BSA was implemented in the buffer to increase the solubility of ligands (Supplementary Materials, Figure S1). In principle, this approach could be exploited to derive the apparent ‘inactivating’ binding IC_50_ values for the antagonist ligands in the presence of an agonist. Thus, we assayed the effects of ligands altering sensor dynamics in the absence and in the presence of BSA.

For rimonabant, we tested the sensor in the various modes using 2-AG (10 and 20 μM) and CP55940 (300 nM) as the activating ligand in the presence and absence of BSA (Figure 2B–D) [43]. In our 96-well plate reader assay using the HEK293 cells, the potency of rimonabant on the GRAB_eCB2.0_ was largely not affected by the presence of BSA in the buffer. The IC_50_ values were remarkably consistent for rimonabant using various experimental agonists with and without BSA (Figure 2B–D). In the presence of 300 nM CP55940, rimonabant suppressed the increase in fluorescence caused by CP55940 on GRAB_eCB2.0_ modified CB_1_ receptors, resulting in a calculated IC_50_ of 31 nM reflecting its antagonist potency. It can be stated that, for this compound, there is valid qualitative and quantitative data proving the fidelity of this sensor. Rimonabant was also tested to antagonize 2-AG, a weaker agonist in comparison to CP55940. Agonist concentrations of 10 μM 2-AG gave IC_50_ of 56 ± 2.3 nM and with 20 μM of 2-AG, an IC_50_ of 181 ± 52 nM was obtained for rimonabant. We then proceeded to test the various known and literature-reported antagonists of cannabinoid CB_1_ receptor. Qualitative data could be derived for all compounds to determine if the modulator is an antagonist, corresponding to its ability to affect the basal fluorescence as well as in the presence of an agonist. In the presence of a constant concentration of CP55940 (300 nM), known antagonists were added to the cells, leading to an observed suppression of fluorescence emitted by the modified CB_1_Rs. These assays were conducted on a concentration gradient, with the modulator’s concentration ranging from 10^−12^ to 10^−5^ for each compound.

Taranabant, another potent inverse agonist/antagonist for the CB_1_R, produced an IC_50_ value of 205 nM on the GRAB_eCB2.0_. This reported Ki of taranabant is 0.13 nM and it showed robust effects in antagonizing CP55940 actuated fluorescence [41,44]. Otenabant is another highly potent antagonist for the CB_1_R that was in clinical trials [45,46]. Similar to taranabant, otenabant was under development as an anti-obesity agent, but clinical trials were halted when rimonabant’s malaise-inducing effects came into light. Otenabant has a reported IC_50_ of 13.1 nM with a binding Ki of 0.7 nM for CB_1_R [46]. The GRAB_eCB2.0_ sensor gave a qualitative curve showing the antagonistic effects of the compound in the assay, also giving an IC_50_ of 27 nM in the presence of 300 nM CP55940.

The GRAB_eCB2.0_ sensor showed robust antagonist responses for prototypical CB_1_ antagonists, with taranabant being an exception. Thus, the sensor can be used with novel compounds when testing for the effect the synthesized modulators on the CB_1_ receptor. Upon addition of BSA, the IC_50_s of the compounds improved for taranabant but surprisingly not for rimonabant and otenabant (Table 1). Taranabant improved to 23 nM IC_50_ in response to the BSA inclusion in the buffer compared to the original 205 nM IC_50_. An IC_50_ value of 41 nM was obtained for otenabant upon the addition of BSA.

### 2.4. 4-Arm Modulators

**Figure 3:**
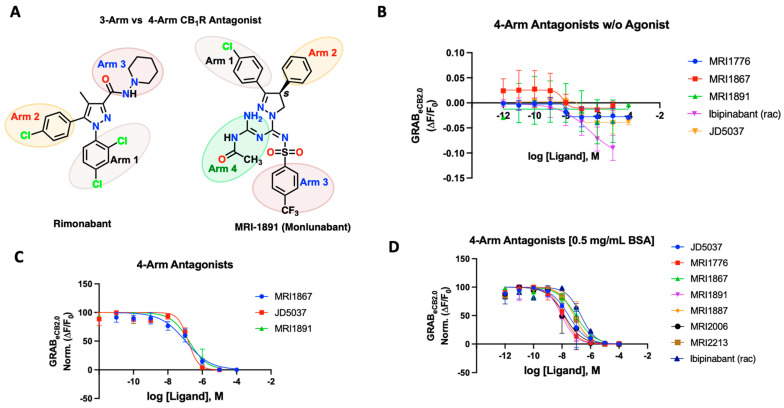
(**A**) Structural depiction of prototypical 3-arm and 4-arm CB_1_R antagonists. (**B**) Fluorescent signal of 4-arm modulators ibipinabant, JD5037, MRI-1867, MRI-1891, and MRI-1776 at inhibiting GRAB_eCB2.0_ basal fluorescence, as determined by averaging ΔF/F_0_ between 4 and 5 min. (**C**) Antagonist potency of select 4-arm ligands JD5037, MRI-1867, and MRI-1891 at inhibiting GRAB_eCB2.0_ fluorescence in presence of 300 nM of CP55940 and in absence of BSA, as determined by averaging ΔF/F_0_ normalized between 4 and 5 min. (**D**) Antagonist potency of select 4-arm modulators ibipinabant, JD5037, MRI-1776, MRI-1867, MRI-1891 MRI-1887, MRI-2213, and MRI-2006 (all compounds tested were enantiomerically pure (*S*)-isomer) at inhibiting GRAB_eCB2.0_ fluorescence in presence of 300 nM of CP55940 with 0.5 mg/mL BSA, as determined by averaging ΔF/F_0_ normalized between 4 and 5 min. IC_50_ values were calculated by GraphPad Prism 9. Data represent mean ± SEM from minimum three independent experiments (*vide infra*, Table 2).

New class of compounds developed in the last decade which function through peripheral mechanisms like JD5037 and, more recently, Zevaquenabant (MRI-1867), Monlunabant (MRI-1891), and MRI-1776. A defining structural feature of these novel compounds is that they belong to the 4-arm antagonist class. In contrast to the previously tested 3-arm antagonist, they contain an additional branch or “arm” arising from the central carbon (Figure 3A). This additional arm can potentially offer unique interactions with certain amino acids in the inactive CB_1_R binding pocket [47,48,49,50,51,52,53]. Our laboratory has been developing such novel, 4-arm, peripherally-restricted antagonists of the CB_1_R, which have shown promise in ameliorating fibrosis of the liver, lung, skin, kidney and treating dyslipidemia, insulin resistance, obesity and attenuating alcohol drinking in rodent models [50,51,52,53,54,55,56,57]. A recent Phase 1b study in patients with metabolic syndrome has shown that MRI-1891/INV-202/Monlunabant causes a significant decrease in body weight and improvement in cardiometabolic parameters without any adverse effects [58]. The 4-arm antagonist developed in our lab provided us an opportunity to test their behavior on the GRAB_eCB2.0_ sensor and their ability to antagonize agonist responses.

We tested various compounds for putative interaction of the fourth arm with the receptor residues and its dynamics upon binding to the receptor. Prototypical compounds JD5037, MRI-1891/Monlunabant, and MRI-1867/Zevaquenabant were tested for their ability to alter sensor dynamics in the presence and absence of CP55940 (Figure 3B–D). JD5037, discovered by Chorvat and colleagues, is a peripherally-restricted analog of the CB_1_R antagonist compound ibipinabant (SLV319) [59]. Ibipinabant has a truncated fourth arm which, upon modification, led to the compound JD5037 via an extended fourth arm conferred by a valinamide moiety that led to a potent antagonism on the CB_1_R with binding Ki = 0.5 nM [59,60]. On the GRAB_eCB2.0_ sensor, the 4-arm antagonists inhibited the fluorescence of the sensor, leading to a concentration-dependent alteration below the baseline, indicative of inverse agonism (Figure 3B). We also tested the antagonists to inhibit agonist fluorescence in the presence of CP55940 (300 nM). JD5037 delivered an IC_50_ of 148 nM, much lower than that of the reported value. MRI-1867 is another reported CB_1_R antagonist with a functional potency IC_50_ of 40 nM and binding Ki of 2.6 nM; the GRAB-based sensor gave a much lower IC_50_ of 105 nM. MRI-1891 is also a potent 4-arm CB_1_R antagonist with a bias toward inhibition of the β-arrestin pathway, over G-protein signaling pathway (IC_50_ = 6 nM) and binding Ki = 0.5 nM. When applied to the GRAB_eCB2.0_, MRI-1891 yielded an IC_50_ of 168 nM. Of note, the 4-arm modulators clearly revealed their ability to reverse the increase in CP55940-mediated fluorescence and behave as antagonists, highlighting the utility of the GRAB_eCB2.0_ to differentiate the functional character of the test compounds (Figure 3C). As previously seen, all tested 4-arm antagonists benefited significantly from the addition of the BSA in the buffer (Figure 3D). MRI-1867 improved to 90 nM, MRI-1891’s IC_50_ on the sensor improved to 9.8 nM, and finally JD5037 improved to 29 nM in the presence of the same agonist-concentration (Table 2). The sensor enabled us to derive apparent potencies of additional 4-arm antagonists that were previously reported, as reflected in Figure 3D (Table S1, Supplementary Materials). In general, the binding potencies trended toward the lower range on the sensor, in comparison to the reported affinities using the wild-type CB_1_R or membrane preparation and were comparable to the functional potencies reported for the compounds. The tested compounds were mainly enantiomerically pure *S*-isomers which were reported earlier to have single-digit nanomolar binding affinities (Table S1, Supplementary Materials) [47,49,50,51,52,53]. The CB_1_ active enantiomer (*S*)- and (*R*)- enantiomer (inactive) could be clearly distinguished on the sensor. For example, the *R*-enantiomers of MRI-1891 and MRI-1776 were reported to have weak CB1 binding and upon testing on the sensor, this was clearly the case (Supplementary Materials, Figure S2).

### 2.5. GRAB_eCB2.0_ Assays for Allosteric Modulators

**Figure 4:**
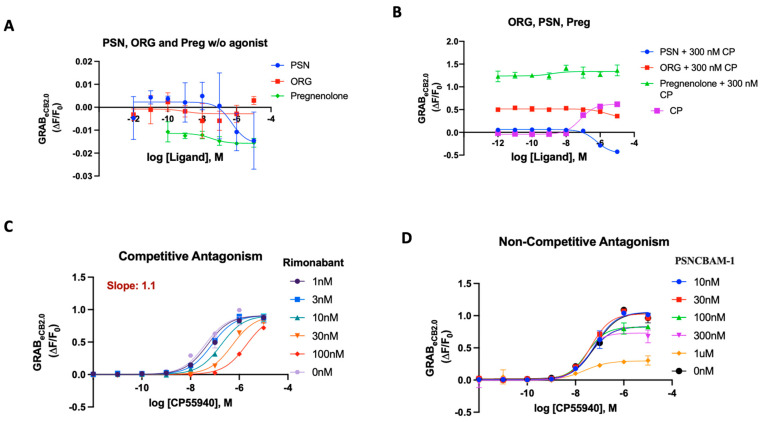
(**A**) Fluorescent signal of ORG, PSN and Pregnenolone at inhibiting GRAB_eCB2.0_ basal fluorescence as determined by averaging ΔF/F_0_ between 4 and 5 min. (**B**) Antagonist potency of ORG PSN and Pregnenolone at inhibiting GRAB_eCB2.0_ fluorescence in presence of 300 nM of CP55940; CP activation of the sensor for reference as determined by averaging ΔF/F_0_ between 4 and 5 min. (**C**) Competitive antagonism of rimonabant in the presence of a range of concentrations of CP55940. Curves were fitted to a nonlinear regression model with four parameters, variable for individual concentrations. Data for rimonabant were used to generate a Schild plot. On application of the Gaddum–Schild equation, a gradient/slope of 1.13 was obtained. (**D**) In presence of a range of concentrations of CP55940, differing from rimonabant, PSN did not significantly affect the EC_50_ value of CP55940. However, the fluorescent response of CP55940 was reduced in a concentration-dependent manner, indicating non-competitive antagonism. EC_50_/IC_50_ values were calculated by GraphPad Prism 9. Data represent mean of minimum three independent experiments.

Allosteric modulation is considered one pathway to alter CB_1_R signaling, while minimizing deleterious side effects associated with direct orthosteric interaction [61,62]. To broaden the pharmacological characterization of GRAB_eCB2.0_ using the 96-well plate reader assay, we sought to investigate the dynamics of the literature-reported allosteric compounds on the sensor [63,64,65]. Stella and colleagues reported the characteristics of cannabidiol (CBD), which acts as a CB_1_R negative allosteric modulator (NAM) on the sensor [21]. It was found that increasing concentrations of CBD did not affect basal GRAB_eCB2.0_ fluorescent signal. We sought to investigate various reported synthetic allosteric compounds like ORG27569 (ORG), PSNCBAM-1 (PSN) and pregnenolone analogues for their behavior on the sensor [65]. ORG27569 has been characterized as an allosteric modulator that shows positive cooperativity for CP55940 binding to CB_1_ but paradoxically acts as an antagonist of G-protein coupling [66,67]. PSNCBAM-1 was characterized as a NAM based on detailed pharmacological assays, but it enhances agonist binding [68]. Pregnenolone (Preg) is classified as a signaling specific negative allosteric modulator on the CB_1_R [64]. When applied to the GRAB_eCB2.0,_ while the compounds ORG27569 and Preg did not significantly alter basal fluorescence, PSN showed weak inverse agonism at higher concentrations (Figure 4A). Upon checking their behavior on the sensor in the presence of 300 nM CP55940, all three compounds extinguished the CP-activation differentially; however, their behavior was void of any concentration-dependent effects, in contrast to antagonists like rimonabant (Figure 4B). For PSN and Preg, this behavior could be replicated in the presence of 20 μM 2-AG (Supplementary Materials, Figure S3A–C). In the presence of 300 nM CP55940, a normalized curve of ORG and PSN indicated their behavior as weak antagonists with IC_50_ 787 nM and 567 nM (Supplementary Materials, Figure S3B). Pregnenolone sulfate was also checked for allosteric effects in presence of CP. GRAB_eCB2.0_sensor behavior showed that this compound also extinguished CP fluorescence but showed no concentration-dependent effects (Supplementary Materials, Figure S3D).

Using the optimized assay in a traditional orthosteric manner works well and delivers reliable agonist and antagonist potencies. The sensor was then tested for its ability to determine the difference between competitive and non-competitive antagonism in an assay mirroring the Schild regression analysis [68,69]. Rimonabant’s robust results as an orthosteric antagonist made it a good competitor with minimum variance in the solubility and potency of its effect. To test for the ability to detect competitive binding on the sensor at the orthosteric position, rimonabant and PSNCBAM-1 (individually) were added on the 96-well plate in a Schild-plot manner, as described in Section 3. Despite the lack of signaling attributes in the sensor, the GRAB_eCB2.0_ sensor could derive the receptor’s activity based on fluorescence in the same mechanistic manner as a traditional assay would and could relay the same regression analysis with strong quantitative correlations. We tested rimonabant in an ‘apparent’ Schild plot using varying concentrations of CP55940 against select concentrations of rimonabant (Figure 4C). Rimonabant, a competitive inverse agonist, resulted in a concentration-dependent increase in the EC_50_ of CP55940. The Schild plot for rimonabant provided a gradient/slope of 1.1, indicating competitive reversible antagonism of an orthosteric nature (Figure 4C).

Through detailed [^35^S]-GTPγS and cAMP assays, PSN was characterized as a non-competitive antagonist using a Schild regression analysis [68]. When PSN was tested in our assay with varying concentrations of CP55940, as described above, the eCB2.0 sensor displayed a behavior that pointed to its non-competitive attributes (Figure 4D). Unlike a competitive antagonist, PSN did not significantly affect the EC_50_ value of CP55,940. However, the efficacy/fluorescent response (apparent equivalent of *E*_max_) of CP55,940 was reduced in a concentration-dependent manner. These data provide validation that PSNCBAM-1 can behave as a non-competitive antagonist on the GRAB_eCB2.0_ sensor. 

We also tested the literature-reported positive allosteric modulators (PAMs) of CB_1_R for their ability to influence the sensor. Commercially available compounds GAT228 and GAT229, which are chiral and enantiomers of racemic GAT211 and are both well characterized, were procured. In various biological systems examined, GAT211’s allosteric agonist activity was shown to reside with the *R*-(+)-enantiomer (GAT228), whereas the *S*-(-)-enantiomer (GAT229), which showed no intrinsic activity, is where the PAM activity resided [70].

**Figure 5:**
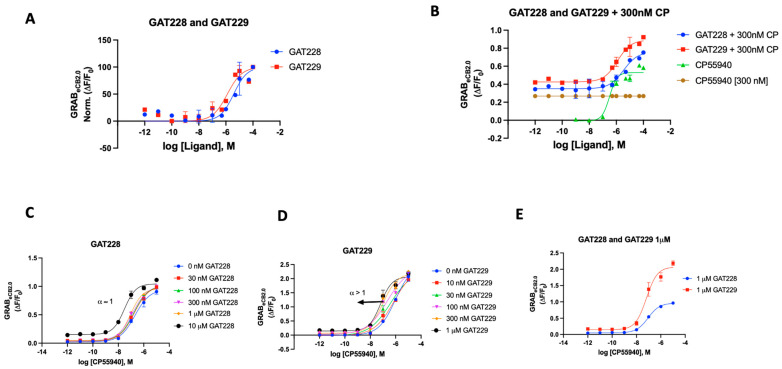
(**A**) Fluorescent signal of GAT228 and GAT229 at altering GRAB_eCB2.0_ basal fluorescence as determined by averaging ΔF/F_0_ normalized between 4 and 5 min. (**B**) Fluorescent signal of GAT228 and GAT229 at altering GRAB_eCB2.0_ fluorescence in presence of 300 nM CP55940, as determined by averaging ΔF/F_0_ between 4 and 5 min. (**C**) Non-competitive agonism of GAT228 in the presence of a range of concentrations of CP55940. Curves were fitted to a nonlinear regression model with four parameters, variable for individual concentrations. (**D**) Non-competitive agonism of GAT228 in the presence of a range of concentrations of CP55940. (**E**) Fluorescent signal of GAT228 and GAT229 (1 μM), altering GRAB_eCB2.0_ fluorescence in presence of varying concentrations of CP55940, as determined by averaging ΔF/F_0_ between 4–5 min. Curves were fitted to a nonlinear regression model with four parameters, with variable slope. EC_50_ values were calculated by GraphPad Prism 9. Data represent mean of minimum three independent experiments.

In the case of the GRAB_eCB2.0_ sensor, both GAT modulators were initially used independently to see if there would be any alteration in the fluorescence signal. Both compounds delivered a weak enhancement of the signal, demonstrating activity as a very weak agonist (EC_50_ 2 and 1 μM, respectively) (Figure 5A). When compared to CP55940, both GAT compounds showed a very weak increase in fluorescence (Supplementary Materials, Figure S4). As previously reported, we then proceeded to test both GAT228 and GAT 229’s ability to interact with the sensor in the presence of CP55940 (300 nM). As shown in Figure 5B, GAT229 was a more potent enhancer of sensor fluorescence than GAT228. This result seemingly agrees with the report that GAT229 does positively modulate the agonist response/fluorescence. GAT228, then, behaves as an allosteric agonist, by virtue of its weak agonism. Upon testing both compounds in Schild-like analyses (Figure 5C,D) to evaluate the positive allosteric effect, we could discern the difference in the characteristics of the curve by their α values. GAT228 had apparent α remaining constant at all concentrations of the agonist, whereas GAT229 had an apparent α > 1. This result aligns with the reported behavior of GAT228 and GAT229, although the sensor does not unambiguously discern the difference in the allosteric subtilities expected with these ligands. An additional detail of the differential effects of the compounds GAT228 and GAT229 could be seen when GAT228 (1 μM) did not shift the fluorescence signal relative to orthosteric ligand CP55940, whereas GAT229 (1 μM) increased the maximal fluorescence induced by CP55,940 in the sensor experimental system.

GRAB_eCB2.0_ was recently designed and reported by Li and colleagues to allow for the detection of change in fluorescent signal in response to changes in endocannabinoid levels in the ECS. Our interest in this sensor was piqued when we envisioned that it could be used as a tool to assay novel cannabinoid modulators. In a simple assay that could potentially offer a ‘hybrid binding-function’ evaluation tool for novel ligands, we sought to develop a method to test functionally indiscriminate CB_1_R modulators. As such, we optimized the sensor system/assay to generate EC_50s_ for compounds that increase the fluorescence with existing agonists so they could be validated on the sensor, as previously reported. For modulators that do not increase the basal fluorescence, we could determine the functional characteristics of the ligands by the compound’s ability to suppress an agonist-induced fluorescence increase in an assay supporting traditional functional assays like [^35^S]-GTPγS or cAMP. The sensor also performed reasonably well to detect previously reported allosteric compounds. Allosteric binding sites on GPCRs are discrete domains in the 7-transmembrane or intracellular regions distinct from orthosteric sites that bind ligands causing a potentiation or attenuation of orthosteric ligand mediated effects and result from changes in protein conformation. Extensive studies have illuminated the NAM effects of PSN ligand. Accordingly, we tested PSN in cell culture in the absence and presence of CP55940. PSN, in the absence of CP, showed no increase above basal fluorescence, while allosteric effects could be detected upon a Schild-like analysis. GAT228 and GAT229, in the presence of 300 nM CP55940, showed allosteric effects mirroring their reported behavior [70]. At the sensor, a distinguishing characteristic of the orthosteric antagonists vs. the allosteric antagonists was the clear concentration-dependent attenuation of agonist fluorescence that was absent in allosteric compounds. In principle, this agrees with the fact that allosteric compounds can affect orthosteric ligand binding from sites far away from the traditional putative binding pocket, and their responses vary based on the ligand in use. This is an area that will require additional evaluation and optimization of the assay parameters to study and validate novel allosteric modulators; the sensor construct devoid of signaling components may limit the study of intricate allosteric mechanisms involved.

As discussed before, the CB_1_ modulators are highly lipophilic compounds which may pose challenges with solubility to dissolve readily in the aqueous buffer. This does seem to influence IC_50_ values, which initially were lower than reported. BSA dissolved in the DPBS aqueous buffer at a concentration of 0.5 mg/mL, increasing the solubility of the lipophilic modulators and resulting in increased congruence to reported EC_50_/IC_50_ values for many of the modulators. Of note, the agonist ligands were tested for their ability to affect the sensor signal in the presence of BSA. This resulted in a larger variance from the reported EC_50_ values (Supplementary Materials, Figure S5).

An additional factor that can alter the sensor performance for certain compounds is the amino acid mutations in the sensor construct. This was particularly evident in cases of the antagonists containing a fourth arm. The binding affinity of the fourth arm with the mutated amino acids plays a role in this differentiation and the hydrophobicity of the molecule itself. 2-AG and rimonabant-like 3-arm antagonists are purported to interact with certain amino acids within the orthosteric binding site in CB1R that are likely present in GRAB_eCB2.0_. The optimized GRAB_eCB2.0_sensor has a serine-to-threonine mutation at the 383rd residue (S383T) and a phenylalanine-to-alanine mutation at the 177th position (F177A). These mutations were induced in the sensor CB_1_R to increase the fluorescence of the attached GFP and allow for better detection. The fourth arm in compounds MRI-1891 and MRI- 1776 were proposed to have interaction with amino acid residues S383 and F177 [49,51,52,53]. Mutated amino acids in the sensor region that are not present in native CB_1_R may contribute to subtle change in binding dynamics and hence, varied IC_50_s could be seen for such compounds using this sensor. The steric features of the arm and the functional groups may further play a role in the interaction and the activity of the receptors in such modulators.

## 3. Materials and Methods

Chemicals and Reagents: 2-AG (Cayman, Ann Arbor, MI, USA #62160), CP55940 (Cayman #13608), ACEA (Biotechne, Minneapolis, MN, USA: Tocris #1319), SR141716 (MedChem Express, New York, NY, USA: #HY14136), Taranabant (MedChem Express #HY10013), Otenabant (MedChem Express #HY-10871), Ibipinabant (MedChem Express HY-14791), and GAT228 and GAT229 (MilliporeSigma: Burlington, MA, USA #SML1952 and #SML1951). ORG27569 (Tocris #2957), PSNCBAM-1 (Cayman #25855), Pregnenolone and Pregnenolone sulfate (Thermo Fisher, Waltham, MA, USA: #AC164420050 and # 537650), JD5037, MRI-1867, MRI-1891, MRI-1776, MRI-1887, MRI-2213, MRI-2594, MRI-2687, and MRI-2006 (structures of compounds in Supplementary Materials, Table S1) were synthesized in-house according to previously published procedures [49,50,51,52,53,59,60]. 

Stable eCB2.0 sensor cell line: A stable sensor cell line was established in HEKtsA201 cells (Sigma-Aldrich, St. Louis, MO, USA) using the PiggyBac Transposon-based system (SBI, Palo Alto, CA, USA). Briefly, the open reading frame of the eCB2.0 sensor from eCB2.0 to C1^20^ was cloned into the PB514B shuttle vector using In Fusion cloning (Takara, San Jose, CA, USA). The modified shuttle sequence was obtained with Sanger sequencing (Psomagen, Rockville, MD, USA) and verified (MacVector, Apex, NC, USA). HEKtsA201 cells were maintained in DMEM (Thermo Fisher, Waltham, MA, USA: Gibco #10569-010) supplemented with 10% fetal bovine serum (Gibco #A31605-01) and 1% penicillin/streptomycin (Gibco #15140-122) at 37 °C and 8% CO_2_. Cells were plated on 12-well tissue culture plates at approximately 50% confluency. This low cell density was used to avoid overgrowth of the cells during the delay between transfection and selection for stable integration. After 3 h, the eCB2.0 shuttle was co-transfected at a plasmid mass ratio of 10:1 with the SBI Transposase vector (PB210PA) using deacylated PEI [71]. All transfected wells were checked the next day for expression of the sensor by presence of basal cpeGFP fluorescence. Sensor expression produced weak eGFP signal as expected; however, the PB514B shuttle carries an RFP (Red Fluorescent Protein) open reading frame driven off a separate Puromycin resistance expression cassette. Hence RFP reporter expression was also monitored. Wells were selected for integration beginning at 4 days post transfection with 10 mg/mL Puromycin (Gibco #A11138-03). Selection was continued throughout all passages of the stable line. Cells were transferred to T25 (25 cm^2^) tissue culture flasks after 10 to 14 days of selection.

**Assay Procedure:** The DMEM solution was removed from the T25 flat-sided cell culture flask by decantation. A total of 3 mL of TrypLE (Gibco #12604-021) was added and allowed to incubate for 2–3 min at 25 °C in a laminar flow hood. A total of 7 mL of fresh DMEM was added, and cells were gently pipetted to remove any remaining cells from the flask wall. The total volume (10 mL) was transferred to a 15 mL Falcon tube and centrifuged at 800× *g* for 4 min at 23 °C. Upon completion of centrifugation, the supernatant was removed and discarded, leaving behind a small pellet of cells that were resuspended in 4 mL of fresh DMEM. The cell suspension was mixed in a 1:1 ratio with 0.4% TrypanBlue (10 mL each) and 10 mL of the combined solution was added to each side of a Countess Cell Counting Chamber and inserted into the Countess II Automated Cell Counter (Thermo Fisher, Waltham, MA, USA). The concentration of cells as adjusted to 0.7–1.0 million cells/mL with DMEM complete media and 100 mL was added to the desired wells of a clear bottom black plastic tissue culture treated 96-well plate. The plate was incubated at 37 °C and 8% CO_2_ for a 24–48 h period. After incubation, the 100 mL DMEM buffer was removed and replaced with 100 mL of DPBS buffer containing calcium and magnesium (Gibco #14040-133). After the addition of the DPBS buffer, a baseline reading of the cells was taken using the PHERAstar FSX microplate reader (BMG/Labtech, Cary, NC, USA) to establish the F0 or initial fluorescence (see Figure 1A,B).

When running the assay for an agonist, 25 mL of the compound and 25 mL of additional DPBS buffer was added to the wells. A concentration curve was made for the modulator with the final concentrations in the wells holding the cells ranging from 1 pM to 10 mM with 1% DMSO BSA in a total volume of 150 mL. The procedure was reapplied to check for EC_50_ values upon inclusion of 0.5 mg/mL BSA in the buffer. The procedure was modified for compounds that did not show an increase in fluorescence upon testing (presumed antagonist/allosteric modulators). A total of 25 μL of the compound and 25 μL of additional DPBS buffer or 25 μL of a standardized agonist (CP55940 or 2-AG) were used to assess fluorescence alterations in the presence and absence of an agonist. A concentration curve was made for the modulator with the final concentration in the wells holding the cells ranging from 1 pM to 10 μM, and the final concentration of the CP55940 in the well was 300 nM (10 or 20 μM if 2-AG) with 1% DMSO and 0.5 mg/mL BSA in a total volume of 150 μL. In either assay, the cannabinoid ligands were added, followed by incubation of the compound on the cells at 37 °C for 5 min before taking a fluorescence reading using the PHERAstar FSX microplate reader equipped with a GFP filter cube module (Ex. 485 nm/Em. 520 nm). The recorded values were the F or final fluorescence.

For reported allosteric modulators, the binding assay was modified to test the ligand for basal activity, followed by the addition of the agonist, similar to the antagonist assay. To assess competitive or non-competitive antagonism, the sensor was tested in an assay akin to the Schild analysis [69,72,73]. The concentration of CP55940 varied from concentrations of 1 pM to 10 mM. The compound of interest was varied across concentrations ranging (as the case may be) from 1 nM, 3 nM, 10 nM, 30 nM, 100 nM, 300 nM, 1 μM, and 10 μM and performed in triplicate. The concentration of the compound depended on the modulator itself. The concentrations calculated were the final concentration inside the well of the 96-well plate, along with the 1% DMSO and 0.5 mg/mL BSA (See Supplementary Materials, Figure S1). A 5 min incubation time was sufficient for detection of peak assay activity as any earlier time points had not reached maximum intensity, and all time points after 5 min show a sustained plateau (See Figure 1B).

**Statistical Methods**: All GRAB_eCB2.0_ fluorescent signals (in cases noted) are expressed as normalized Δ*F/F*_0_ as calculated for the fluorescence assay in the PHERAstar FSX Plate reader. EC_50_/IC_50_ values were calculated using GraphPad Prism 9 by fitting data to a nonlinear regression model with normalized variable slope (four parameters, normalized where indicated) or using three-parameter response. Data represent mean ± SEM from a minimum of at least three independent experiments performed in triplicates.

## 4. Conclusions

In an approach amenable to a high-throughput or 96-well plate reader assay platform in HEK293 cells, we show that 2-AG, CP55940, WIN5512, ACEA, MRI-2594, and MRI-2687 increase the GRAB_eCB2.0_ fluorescent signal and behave as agonists of CB_1_R. However, the extent of the increase in fluorescence differs with EC_50_ values that are modestly comparable to wildtype CB1R estimated radioligand binding. Expanding on the utility of the GRAB_eCB2.0,_ we also observed that rimonabant dose-dependently blocks the agonist mediated increase in the GRAB_eCB2.0_ fluorescent signal, with an IC_50_ in line with its reported potency at CB_1_R. In the absence of an agonist, rimonabant’s effect modulating the sensor could be measured at levels below baseline fluorescent signal, suggesting inverse agonism. This effect was indeed seen for other tested antagonists, including 4-arm ligands. Various antagonists were validated with the sensor for their ability to antagonize the agonist-induced fluorescence increase, supporting the use of GRAB_eCB2.0_ as a pharmacological tool. PSN modulated the CP-induced increase in the GRAB_eCB2.0_ fluorescent signal but not its basal fluorescent signal significantly. In a method mimicking Schild analysis, PSN’s behavior indicated that the non-competitive molecular mechanism seen in native CB_1_R is maintained in GRAB_eCB2.0_. In similar experiments, rimonabant unambiguously behaved as a competitive, orthosteric antagonist. Indeed, rimonabant behaved as a tool compound with high fidelity on the sensor, further attesting to its utility as a control for sensor-based experiments in the current model system. Allosteric behavior using PAMs were also gleaned on the sensor. Thus, GRAB_eCB2.0_ provides an opportunity to study changes in the binding dynamics of NAMs, PAMs and functionally disparate CB_1_ ligands belonging to varied chemotypes in a robust in vitro cell culture system. Our results delineate and validate the pharmacological profiles and binding dynamics of cannabinoid receptor ligands using a GRAB_eCB2.0_ sensor system and offer opportunities for improving the sensor technology to ‘sense’ subtle changes induced by ligands with privileged and yet-to be explored chemotypes targeting the endocannabinoid CB_1_ receptor in a simple model platform. Further validation of the sensor should be possible through future studies on novel ligands with varying cannabinoid receptor functional profiles.

## Figures and Tables

**Table 1:** Effect of 3-arm antagonists in modulating GRAB_eCB2.0_ fluorescence in presence of 300 nM CP55940.

Compound	GRAB_eCB2.0_ IC_50_ [nM]	GRAB_eCB2.0_ IC_50_ [nM] 0.5 mg/mL BSA
Rimonabant	31 ± 9.5	25 ± 2.5
Taranabant	205 ± 15	23 ± 5
Otenabant	27 ± 7.5	41 ± 4.8

**Table 2:** Antagonism IC_50_ values of prototypical 4-arm modulators on GRAB_eCB2.0_ in the absence of BSA and in the presence of 0.5 mg/mL BSA.

Compound	GRAB_eCB2.0_ IC_50_ [nM]	GRAB_eCB2.0_ IC_50_ [nM] 0.5 mg/mL BSA
MRI-1867	105 ± 5.4	90 ± 5
MRI-1891	168 ± 57	9.8 ± 1.4
JD5037	148 ± 12	29 ± 3.5

## Data Availability

Data are contained within the article.

## Supplementary Materials

The supporting information can be downloaded at: https://www.mdpi.com/article/10.3390/ijms25095012/s1. Reference [74] are cited in the supplementary materials.
