# Supplementary material for: Pharmacological Evaluation of Cannabinoid Receptor Modulators Using GRABeCB2.0 Sensor
Source: Int J Mol Sci. 2024 May 3;25(9):5012. doi: 10.3390/ijms25095012 (PMC11084632; doi:10.3390/ijms25095012)
Supplement: Supplementary file 1 [file ijms-25-05012-s001.zip › ijms-2886307-supplementary.pdf]

Pharmacological Evaluation of Cannabinoid Receptor Modulators Using GRAB$_{eCB2.0}$ Sensor

Samay Shivshankar,[1] Josephine Nimely,[1] Henry Puhl III[2] and Malliga R. Iyer[1]

[1]Section on Medicinal Chemistry, National Institute on Alcohol Abuse and Alcoholism, National Institutes of Health, 5625 Fishers Lane, Rockville, MD 20852, USA.

[2]Laboratory of Biophotonics and Quantum Biology, National Institute on Alcohol Abuse and Alcoholism, National Institutes of Health, 5625 Fishers Lane, Rockville, MD 20852, USA.

Correspondence: malliga.iyer@nih.gov

ORCID: 0000-0002-0116-4619

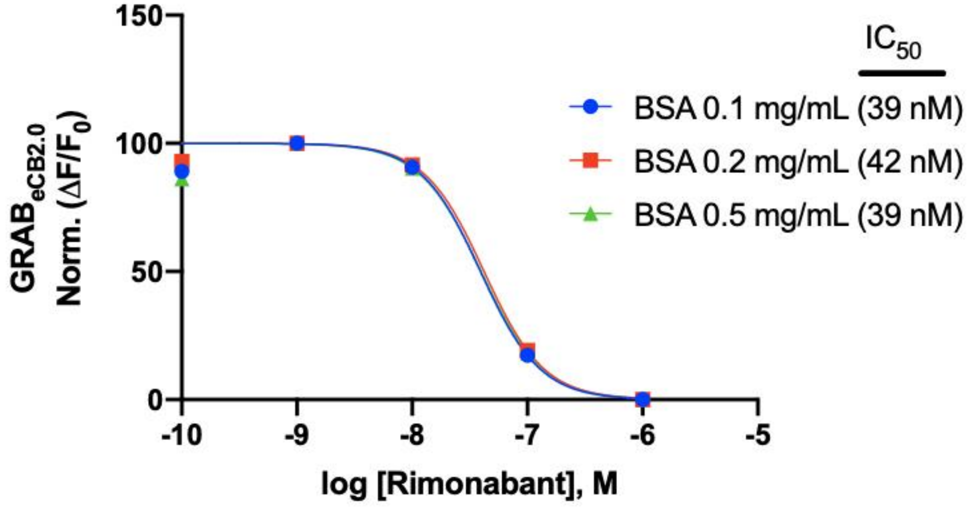

Figure 1. Rimonabant antagonist curve with 300 nM CP55940 as an agonist on the eCB2.0 sensor as normalized. The concentration curve runs from 10^{-10} to 10^{-6} at varied BSA concentrations (0.1 -0.5 mg/mL) of final volume. Curves were fitted to a nonlinear regression model with 4 parameter, variable slope. IC_{50} values were calculated by GraphPad Prism 9. Data represent mean of minimum three independent experiments.

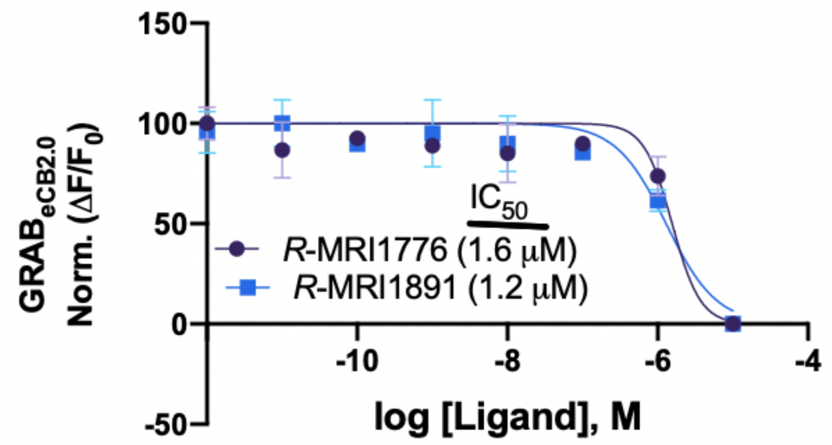

Figure 2. A. Antagonist potency of *R*-MRI-1776 and *R*-MRI-1891 at inhibiting $GRAB_{eCB2.0}$ fluorescence in presence of 300 nM of CP55940 as determined by averaging $\Delta F/F_0$ normalized between 4-5 min. Curves were fitted to a nonlinear regression model with 4 parameter, variable slope. IC_{50} values were calculated by GraphPad Prism 9. Data represent mean of minimum three independent experiments.

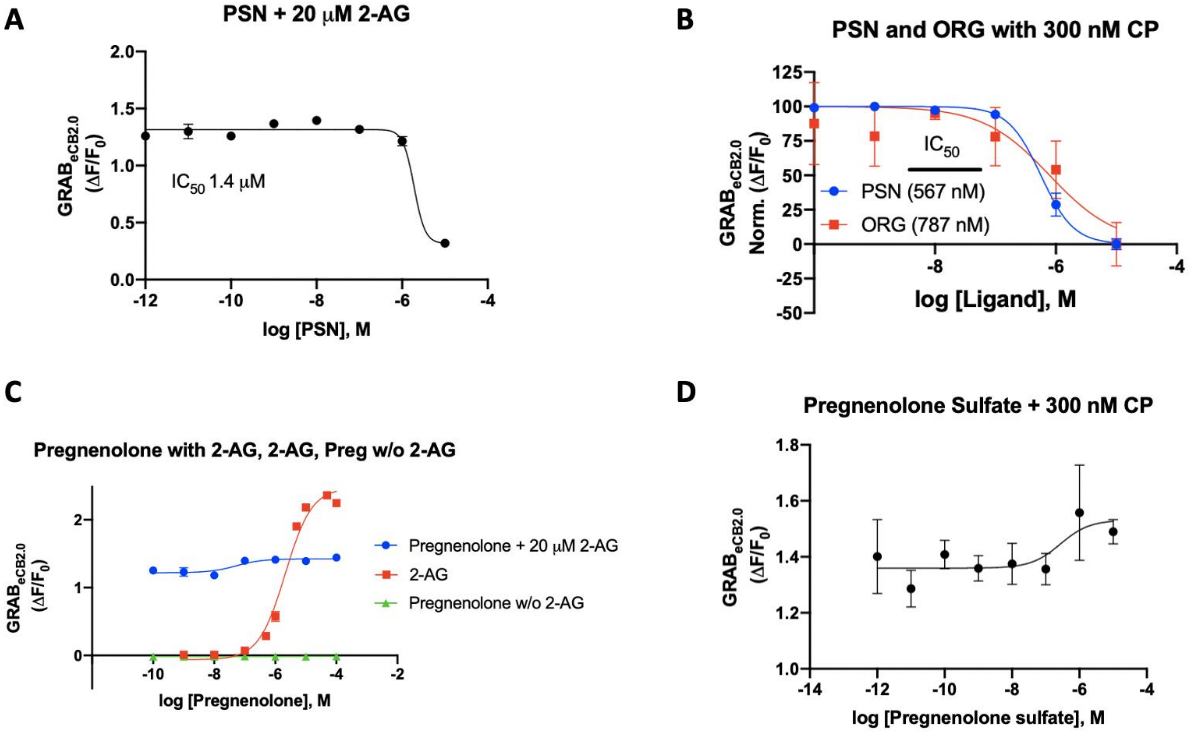

Figure 3. A. Fluorescent signal of PSN in the presence of 20 μM 2-AG at inhibiting $GRAB_{eCB2.0}$ fluorescence as determined by averaging $\Delta F/F_0$ between 4-5 min. **B.** Fluorescent signal of PSN and ORG in the presence of 300 nM CP55940 at inhibiting $GRAB_{eCB2.0}$ fluorescence as determined by averaging $\Delta F/F_0$ and normalizing between 4-5 min. **C.** Fluorescent signal of Pregnenolone in the presence and absence of 20 μM 2-AG at inhibiting $GRAB_{eCB2.0}$ fluorescence as determined by averaging $\Delta F/F_0$ between 4-5 min. **D.** Fluorescent signal of Pregnenolone sulfate in the presence of 300 nM CP55940 at inhibiting $GRAB_{eCB2.0}$ fluorescence as determined by averaging $\Delta F/F_0$ between 4-5 min. Curves were fitted to a nonlinear regression model with 3-parameter, variable by GraphPad Prism 9. Data represent mean of three independent experiments.

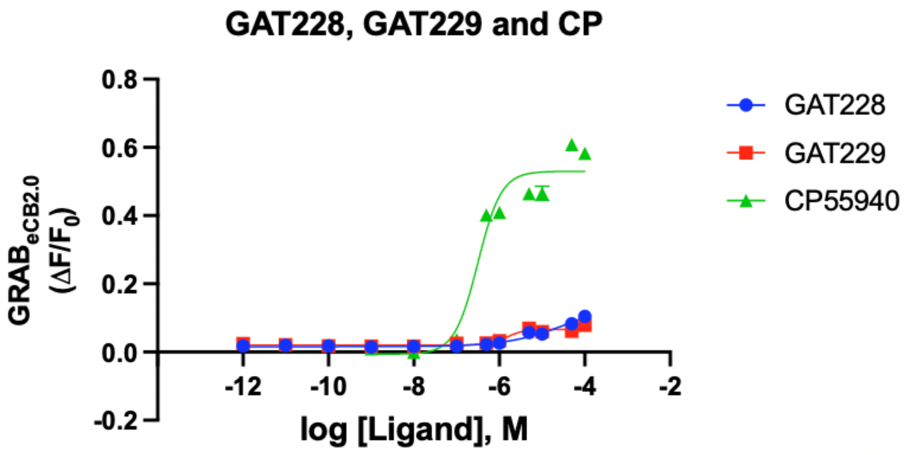

Figure 4. Concentration dependent responses of GAT228 and GAT229, and CP55940 at inducing $GRAB_{eCB2.0}$ fluorescent signal in the presence of 0.5 mg/mL BSA as determined by averaging $\Delta F/F_0$ between 4-5 min. Data represent mean from minimum three independent experiments.

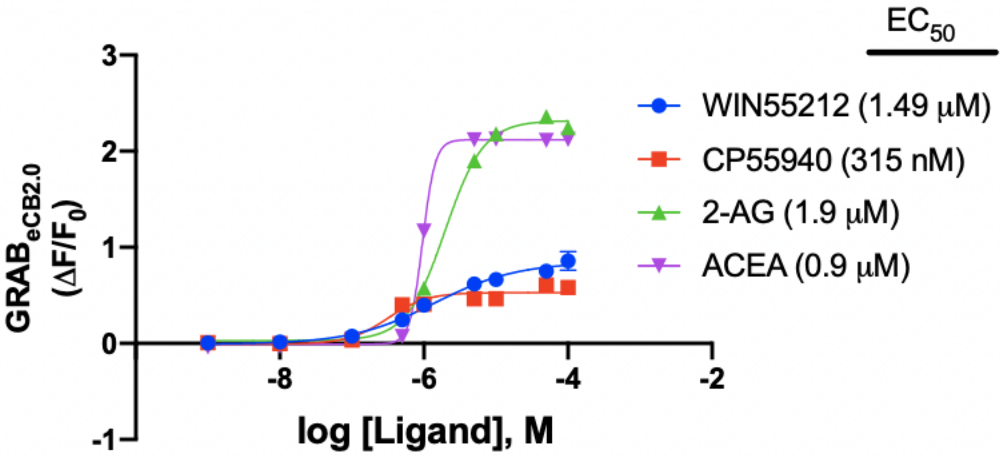

Figure 5. Concentration dependent responses and EC_{50} of 2-AG, ACEA, CP55940, and WIN55212-2, at inducing $GRAB_{eCB2.0}$ fluorescent signal in the presence of 0.5 mg/mL BSA as determined by averaging $\Delta F/F_0$ between 4-5 min. EC_{50} values were calculated by GraphPad Prism 9. Data represent mean from minimum three independent experiments.

Table 1.

**Structure**	**$GRAB_{eCB2.0}$$^{*\&}$ EC_{50} [nM]**	**CB1R wild-type binding# K_i[nM]**
2-((1*R*,2*R*,5*R*)-5-hydroxy-2-(3-hydroxypropyl)cyclohexyl)-5-(2-methyloctan-2-yl)phenol 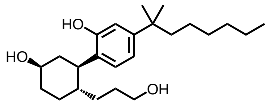 CP55,940	$64 \pm 8^*$ $(315)^{\&}$	0.6-5
1,3-dihydroxypropan-2-yl (5*Z*,8*Z*,11*Z*,14*Z*)-icosa-5,8,11,14-tetraenoate 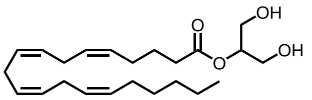 2-AG	$2000 \pm 296^*$ $(1900)^{\&}$	25-472
(5*Z*,8*Z*,11*Z*,14*Z*)-*N*-(2-chloroethyl)icosa-5,8,11,14-tetraenamide 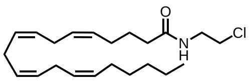 ACEA	$572 \pm 4.1^*$ $(900)^{\&}$	1.4

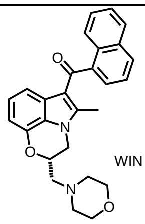<br>WIN 55,212-2<br>(*S*)-(5-methyl-2-(morpholinomethyl)-2,3-dihydro-[1,4]oxazino[2,3,4-*hi*]indol-6-yl)(naphthalen-1-yl)methanone	$564 \pm 34^{*}$<br>$(1490)^{\&}$	62.3
(*Z*)-2-(4,5-dimethyl-2-((2,2,3,3-tetramethylcyclopropane -1-carbonyl)imino)thiazol-3(2*H*)-yl)ethyl acetate<br>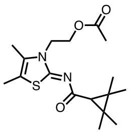<br>MRI-2594	$527 \pm 27^{*}$<br>$(25 \pm 27.5)^{\&}$	[1]
2-(6-methyl-2-((2,2,3,3-tetramethylcyclopropane -1-carbonyl)imino)benzo[*d*]thiazol-3(2*H*)-yl)ethyl acetate<br>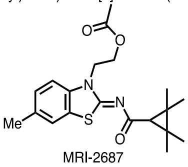<br>MRI-2687	$1300 \pm 182^{*}$<br>$(25 \pm 27.5)^{\&}$	-[1]
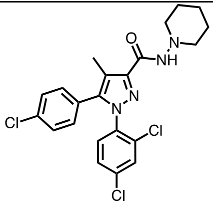<br>Rimonabant	$25 \pm 2.5^{\&}$	5.6[2]
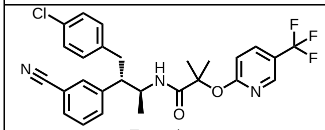<br>Taranabant	$23 \pm 5^{\&}$	0.13[3]
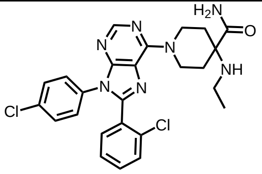<br>Otenabant	$41 \pm 4.8^{\&}$	0.7[4]
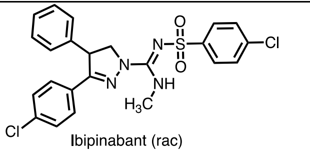<br>Ibipinabant (rac)	$214 \pm 36^{\&}$	7.8[5]
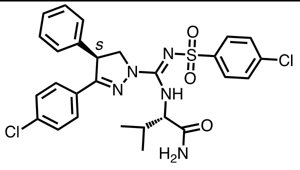<br>JD5037	$29 \pm 3.5^{\&}$	0.5[6]

N-(-*N'*-((*S*)-3-(4-chlorophenyl)-4-phenyl-4,5-dihydro-1*H*-pyrazol-1-yl) (((4-(trifluoromethyl)phenyl)sulfonyl)imino)methyl)carbamimidoyl)acetamide<br>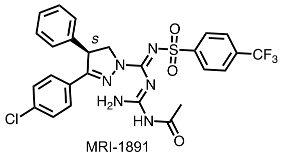<br>MRI-1891	9.8 ± 1.4[&]	0.3[7]
(*S*)-*N*-((*Z*)-1-aminoethylidene)-3-(4-chlorophenyl)-4-phenyl-*N'*-((4-(trifluoromethyl)phenyl)sulfonyl)-4,5-dihydro-1*H*-pyrazole-1-carboximidamide<br>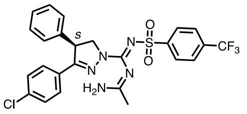<br>MRI-1867	90 ± 5.4[&]	2.3[8,9]
N-(-*N'*-(-((*S*)-3-(4-chlorophenyl)-4-phenyl-4,5-dihydro-1*H*-pyrazol-1-yl)(((4-chlorophenyl)sulfonyl)imino)methyl)carbamimidoyl)acetamide<br>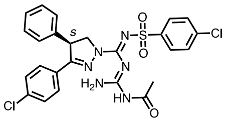<br>MRI-1776	13 ± 1.8[&]	0.81[10]
MRI1887	95 ± 6.5[&]	0.85[10]
MRI2006	36 ± 3.1[&]	0.5[10]
(*S*)-*N*-1-aminoethylidene)-3-(4-chlorophenyl)-*N'*-(*N*,*N*-diethylsulfamoyl) -4-phenyl-4,5-dihydro-1*H*-pyrazole-1-carboximidamide<br>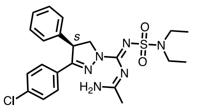<br>MRI-2213	69 ± 1.2[&]	1.2[11]
N-(-*N'*-((*R*)-3-(4-chlorophenyl)-4-phenyl-4,5-dihydro-1*H*-pyrazol-1-yl) (((4-(trifluoromethyl)phenyl)sulfonyl)imino)methyl)carbamimidoyl)acetamide<br>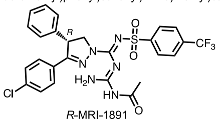<br>*R*-MRI-1891	1600[&]	N.A
N-(-*N'*-(-((*R*)-3-(4-chlorophenyl)-4-phenyl-4,5-dihydro-1*H*-pyrazol-1-yl)(((4-chlorophenyl)sulfonyl)imino)methyl)carbamimidoyl)acetamide<br>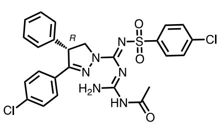<br>*R*-MRI-1776	1200[&]	105[10]
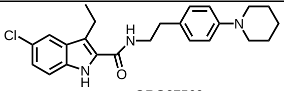<br>ORG27569	787[&]	217[12]
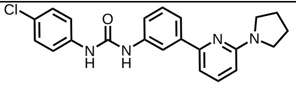<br>PSNCBAM-1	567[&]	45-230[13]

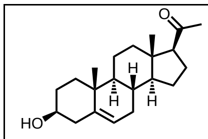 Pregnenolone	-	-
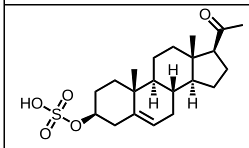 Pregnenolone sulfate	-	-
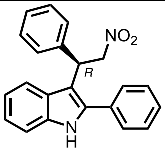 GAT228	&	-
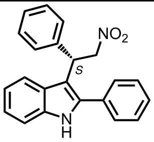 GAT229	&	358[14]

*Values ligand binding experiments carried out without BSA in the buffer. Of note, the agonists performed better on the eCB2.0 sensor in the absence of BSA in buffer.
[&]Values ligand binding experiments carried out without BSA in the buffer. Of note, the antagonists performed better on the eCB2.0 sensor in the presence of 0.5 mg/mL BSA in buffer.

[#]Literature reported values where available.
N.A not available

Bibliography

(1) Li, X.; Hua, T.; Vemuri, K.; Ho, J.-H.; Wu, Y.; Wu, L.; Popov, P.; Benchama, O.; Zvonok, N.; Locke, K.; Qu, L.; Han, G. W.; Iyer, M. R.; Cinar, R.; Coffey, N. J.; Wang, J.; Wu, M.; Katritch, V.; Zhao, S.; Kunos, G.; Bohn, L. M.; Makriyannis, A.; Stevens, R. C.; Liu, Z.-J. Crystal Structure of the Human Cannabinoid Receptor CB2. *Cell* **2019**, *176*, 459–467.e13.

(2) Rinaldi-Carmona, M.; Pialot, F.; Congy, C.; Redon, E.; Barth, F.; Bachy, A.; Brelière, J. C.; Soubrié, P.; Le Fur, G. Characterization and Distribution of Binding Sites for [3H]-SR 141716A, a Selective Brain (CB1) Cannabinoid Receptor Antagonist, in Rodent Brain. *Life Sci.* **1996**, *58*, 1239–1247.

(3) Lin, L. S.; Lanza, T. J.; Jewell, J. P.; Liu, P.; Shah, S. K.; Qi, H.; Tong, X.; Wang, J.; Xu, S. S.; Fong, T. M.; Shen, C.-P.; Lao, J.; Xiao, J. C.; Shearman, L. P.; Stribling, D. S.; Rosko, K.; Strack, A.; Marsh, D. J.; Feng, Y.; Kumar, S.; Samuel, K.; Yin, W.; Van der Ploeg, L. H. T.; Goulet, M. T.; Hagmann, W. K. Discovery of N-[(1S,2S)-3-(4-Chlorophenyl)-2- (3-Cyanophenyl)-1-Methylpropyl]-2-Methyl-2- {[5-(Trifluoromethyl)Pyridin-2-Yl]Oxy}propanamide (MK-0364), a Novel, Acyclic Cannabinoid-1 Receptor Inverse Agonist for the Treatment of Obesity. *J. Med. Chem.* **2006**, *49*, 7584–7587.

(4) Griffith, D. A.; Hadcock, J. R.; Black, S. C.; Iredale, P. A.; Carpino, P. A.; DaSilva-Jardine, P.; Day, R.; DiBrino, J.; Dow, R. L.; Landis, M. S.; O’Connor, R. E.; Scott, D. O. Discovery of 1-[9-(4-Chlorophenyl)-8-(2-Chlorophenyl)-9H-Purin-6-Yl]-4-Ethylaminopiperidine-4-Carboxylic Acid Amide Hydrochloride (CP-945,598), a Novel, Potent, and Selective Cannabinoid Type 1 Receptor Antagonist. *J. Med. Chem.* **2009**, *52*, 234–237.

(5) Lange, J. H. M.; Coolen, H. K. A. C.; van Stuivenberg, H. H.; Dijksman, J. A. R.; Herremans, A. H. J.; Ronken, E.; Keizer, H. G.; Tipker, K.; McCreary, A. C.; Veerman, W.; Wals, H. C.; Stork, B.; Verveer, P. C.; den Hartog, A. P.; de Jong, N. M. J.; Adolfs, T. J. P.; Hoogendoorn, J.; Kruse, C. G. Synthesis, Biological Properties, and Molecular Modeling Investigations of Novel 3,4-Diarylpyrazolines as Potent and Selective CB(1) Cannabinoid Receptor Antagonists. *J. Med. Chem.* **2004**, *47*, 627–643.

(6) Chorvat, R. J.; Berbaum, J.; Seriacki, K.; McElroy, J. F. JD-5006 and JD-5037: Peripherally Restricted (PR) Cannabinoid-1 Receptor Blockers Related to SLV-319 (Ibipinabant) as Metabolic Disorder Therapeutics Devoid of CNS Liabilities. *Bioorg. Med. Chem. Lett.* **2012**, *22*, 6173–6180.

(7) Liu, Z.; Iyer, M. R.; Godlewski, G.; Jourdan, T.; Liu, J.; Coffey, N. J.; Zawatsky, C. N.; Puhl, H. L.; Wess, J.; Meister, J.; Liow, J.-S.; Innis, R. B.; Hassan, S. A.; Lee, Y. S.; Kunos, G.; Cinar, R. Functional Selectivity of a Biased Cannabinoid-1 Receptor (CB1R) Antagonist. *ACS Pharmacol. Transl. Sci.* **2021**, *4*, 1175–1187.

(8) Cinar, R.; Iyer, M. R.; Liu, Z.; Cao, Z.; Jourdan, T.; Erdelyi, K.; Godlewski, G.; Szanda,

G.; Liu, J.; Park, J. K.; Mukhopadhyay, B.; Rosenberg, A. Z.; Liow, J.-S.; Lorenz, R. G.; Pacher, P.; Innis, R. B.; Kunos, G. Hybrid Inhibitor of Peripheral Cannabinoid-1 Receptors and Inducible Nitric Oxide Synthase Mitigates Liver Fibrosis. *JCI Insight* **2016**, *1*.

(9) Iyer, M. R.; Cinar, R.; Coffey, N. J.; Kunos, G. Synthesis of 13 C6 -Labeled, Dual-Target Inhibitor of Cannabinoid-1 Receptor (CB1 R) and Inducible Nitric Oxide Synthase (INOS). *J. Labelled Comp. Radiopharm.* **2018**.

(10) Dvorácskó, S.; Herrerias, A.; Oliverio, A.; Bhattacharjee, P.; Pommerolle, L.; Liu, Z.; Feng, D.; Lee, Y.-S.; Hassan, S. A.; Godlewski, G.; Cinar, R.; Iyer, M. R. Cannabinoformins: Designing Biguanide-Embedded, Orally Available, Peripherally Selective Cannabinoid-1 Receptor Antagonists for Metabolic Syndrome Disorders. *J. Med. Chem.* **2023**, *66*, 11985–12004.

(11) Iyer, M. R.; Cinar, R.; Wood, C. M.; Zawatsky, C. N.; Coffey, N. J.; Kim, K. A.; Liu, Z.; Katz, A.; Abdalla, J.; Hassan, S. A.; Lee, Y.-S. Synthesis, Biological Evaluation, and Molecular Modeling Studies of 3,4-Diarylpyrazoline Series of Compounds as Potent, Nonbrain Penetrant Antagonists of Cannabinoid-1 (CB1R) Receptor with Reduced Lipophilicity. *J. Med. Chem.* **2022**, *65*, 2374–2387.

(12) Ahn, K. H.; Mahmoud, M. M.; Kendall, D. A. Allosteric Modulator ORG27569 Induces CB1 Cannabinoid Receptor High Affinity Agonist Binding State, Receptor Internalization, and Gi Protein-Independent ERK1/2 Kinase Activation. *J. Biol. Chem.* **2012**, *287*, 12070–12082.

(13) Horswill, J. G.; Bali, U.; Shaaban, S.; Keily, J. F.; Jeevaratnam, P.; Babbs, A. J.; Reynet, C.; Wong Kai In, P. PSNCBAM-1, a Novel Allosteric Antagonist at Cannabinoid CB1 Receptors with Hypophagic Effects in Rats. *Br. J. Pharmacol.* **2007**, *152*, 805–814.

(14) Laprairie, R. B.; Kulkarni, P. M.; Deschamps, J. R.; Kelly, M. E. M.; Janero, D. R.; Cascio, M. G.; Stevenson, L. A.; Pertwee, R. G.; Kenakin, T. P.; Denovan-Wright, E. M.; Thakur, G. A. Enantiospecific Allosteric Modulation of Cannabinoid 1 Receptor. *ACS Chem. Neurosci.* **2017**, *8*, 1188–1203.
